# Glycosphingolipids (GSLs) from *Sphingomonas paucimobilis* Increase the Efficacy of Liposome-Based Nanovaccine against *Acinetobacter baumannii*-Associated Pneumonia in Immunocompetent and Immunocompromised Mice

**DOI:** 10.3390/molecules27227790

**Published:** 2022-11-12

**Authors:** Masood Alam Khan, Khaled S. Allemailem, Hamzah Maswadeh, Hina Younus

**Affiliations:** 1 Department of Basic Health Sciences, College of Applied Medical Sciences, Qassim University, Buraydah 51452, Saudi Arabia; 2 Department of Medical Laboratories, College of Applied Medical Sciences, Qassim University, Buraydah 51452, Saudi Arabia; 3 Department of Pharmaceutics, College of Pharmacy, Qassim University, Buraydah 51452, Saudi Arabia; 4 Interdisciplinary Biotechnology Unit, Faculty of Life Sciences, Aligarh Muslim University, Aligarh 202002, India

**Keywords:** *Acinetobacter baumannii*, glycosphingolipids, liposomes, immune response

## Abstract

Due to the high propensity of drug resistance in *Acinetobacter baumannii*, the number of currently available therapeutic drugs has become very limited. Thus, it becomes incredibly important to prepare an effective vaccine formulation capable of eliciting an effective immune response against *A. baumannii*. In this study, we prepared a liposomal vaccine formulation bearing glycosphingolipids (GSLs) from *Sphingomonas paucimobilis* and loaded with the whole cell antigen (WCAgs-GSLs-liposomes) of *A. baumannii*. The immune-stimulating potential and prophylactic efficacy of WCAgs-GSLs-liposomes were compared with those of WCAgs-liposomes (without GSLs) or free WCAgs in both immunocompetent and immunodeficient mice. The efficacy of vaccine formulations was determined by analyzing antibody titer, cytokine levels, and survival studies in the immunized mice. The findings revealed that vaccination with WCAgs-GSLs-liposomes stimulated a greater secretion of antibodies and cytokines, higher lymphocyte proliferation, and increased expression of the co-stimulatory molecules. Anti-sera from WCAgs-GSLs-liposomes-immunized mice remarkably reduced the biofilm formation by *A. baumannii*. Most importantly, WCAgs-GSLs-liposomes-vaccinated mice demonstrated a higher defiance against the pathogen, as compared to the immunizations with WCAgs-liposomes (without GSLs) or free WCAgs. Immunocompetent mice immunized with WCAgs-GSLs-liposomes showed a 100% survival rate, while those immunized with WCAgs-liposomes exhibited a 60% survival rate. The protective effect of WCAgs-GSLs-liposomes was also found to be higher in immunocompromised mice, as the immunized mice showed a 50% survival rate, which was greater than the 20% survival rate of those immunized with WCAgs-liposomes. The survival data was also supported by the findings of bacterial load and histological analysis that substantiated the greatest prophylactic potential of the WCAgs-GSLs-liposomes. These findings recommend that WCAgs-GSLs-liposomes may be reckoned as a prospective vaccine to protect the persons against *A. baumannii* infection.

## 1. Introduction

*Acinetobacter baumannii* has been reported as a common pathogen in the healthcare facility environment in recent years [1]. It causes a broad array of infections, including the pneumonia, meningitis, urinary tract, and soft tissue infections [2]. Immunosuppression is considered one of the main risk factors for *A. baumannii*-associated pneumonia accounting about 35–70% mortality [3]. In the Kingdom of Saudi Arabia long, there has been a continuous expansion in the surfacing of multidrug-resistant (MDR) *A. baumannii* [3,4]. The incidence of antibiotic-resistant *A. baumannii* was found to be 80.3% for ceftazidime, 83.6% for ceftriaxone, and 82% for piperacillin-tazobactam [5,6]. Biofilm formation is one of the important strategies of *A. baumannii*, in order to defy therapy and host immunity [7]. It also contributes to the phenomenon of antibiotic resistance in *A. baumannii* [8,9]. The MDR *A. baumannii* poses the foremost challenge to the clinicians because of the persistent nature of the infections. 

Since the treatment options of MDR, *A. baumannii* infections are becoming very challenging and limited; hence, it is critical to formulate an effective vaccine, in order to protect the individuals. Several studies were performed to formulate a useful vaccine against *A. baumannii*, but none of them has progressed to the clinical stage. Multiple antigens, including inactivated whole organism and outer membrane protein A (OmpA)- and DNA-based vaccines, have been exploited in model animals [9]. The Omp A-based subunit vaccine has shown its prophylactic effectiveness against the drug-resistant *A. baumannii* [10,11]. Moreover, the synthesized multi-epitope polypeptide rOmp22 protected mice against *A. baumannii* [12]. DNA vaccines encoding OmpA and Pal antigens have also imparted resistance to mice against the pulmonary *A. baumannii* infection [13]. Recently, a glyco-engineered vaccine was reported to be effective in inducing a protective immune response against *A. baumannii* [14]. 

**Figure 1:**
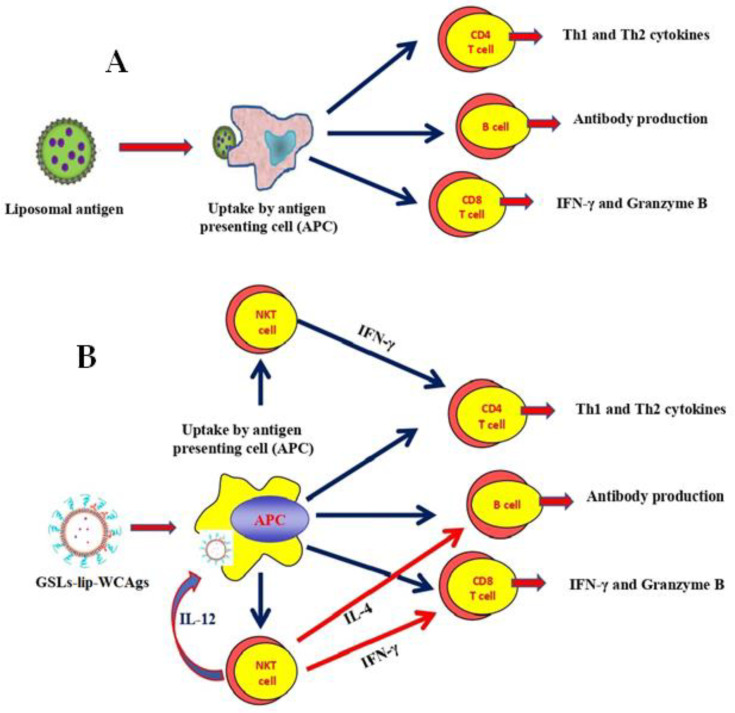
(**A**) Liposomal antigens can induce humoral and cell-mediated immune responses. (**B**) In addition to activating the antibody and cell-mediated immune responses, antigens encapsulated in GSLs-bearing liposomes can induce NKT cell activation that further potentiates immune responses.

Liposomes can act as an effective immunoadjuvant that can activate both the humoral and cell-mediated immune responses (Figure 1A) [15,16]. Liposomes can be prepared by using several types of lipids, including phosphatidylcholine, dipalmitoyl-Sn-glycerol-3-phosphotidylcholine, phosphatidylethanolamine, phosphatidylserine, and sphingomyelin. A liposome-based conjugate vaccine containing monophosphoryl A showed great efficacy against *Campylobacter jejuni* in mice [17]. We have earlier reported that liposomes composed of *Escherichia coli* lipids possess fusogenic potential and potently activate the antigen-reactive cytotoxic T lymphocyte (CTL) response [18]. Conversely, the contamination of lipopolysaccharide (LPS) in *E. coli* lipids may exert some toxicity. Immunization with liposomes containing monophosphoryl lipid A can induce a protective immunity, as liposome-incorporated antigens can be delivered to the antigen processing sites in antigen presenting cells (APCs) that can induce both CD4+ and CD8+ T cells [15]. Alpha-galactosylceramide (α-GalCer) isolated from a marine sponge *Agelas mauritianus* possesses a potent immune-stimulating activity that can be further improved regarding its incorporation in liposomes [15]. *Sphingomonas paucimobilis* is an LPS-free gram-negative bacteria that contains various lipids, including phosphatidylcholine (PC), phosphatidylethanolamine (PE), phosphatidyl monomethyl ethanolamine (PME), phosphatidylglycerol (PG), diphosphatidylglycerol (DPG), and glycosphingolipids (GSLs) [19,20]. GSLs have been demonstrated to stimulate the cells of the innate immune responses, called natural killer T (NKT) cells, that can further activate dendritic cells (Figure 1B) [21,22]. We have earlier shown that the administration of liposomes containing GSLs enhanced the activity of doxorubicin in a mouse model of tumors [23]. In this study, we isolated GSLs from *S. paucimobilis* and prepared a liposomal formulation (GSLs-liposomes) that was used as a vaccine carrier for the *A. baumannii* antigens. The findings demonstrated that immunization with WCAgs-GSLs-liposomes not only elicit higher antigen-specific immune response, but also protect the mice against *A. baumannii* infection. 

## 2. Results

### 2.1. Analysis of the PDI, Size, and Zeta Potential of Liposomes

The characteristics of Lip-WCAgs and GSLs-lip-WCAgs are presented in Table 1.

### 2.2. BMDCs Treated with GSLs-Liposomes Stimulate the Splenocytes to Secrete Higher Levels of IFN-γ

**Figure 2:**
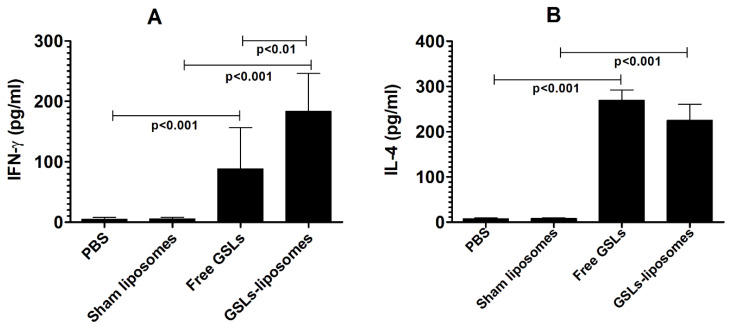
The levels of (**A**) IFN-γ and (**B**) IL-4 in the supernatant of splenocytes co-cultured with GSLs-liposomes. Free GSLs- or GSLs-liposomes-loaded BMDCs were co-cultured with the splenocytes for 48 h, and the culture supernatant was collected to analyze IFN-γ and IL-4. The data are expressed as mean ± SD of three values.

The activating effect of free GSLs or GSLs-liposomes was analyzed by measuring the levels of IFN-γ and IL-4 in the supernatant of splenocytes co-cultured with free GSLs- or GSLs-liposomes-loaded BMDCs. The level of IFN-γ was found to be 183.3 ± 25.2 pg/mL in the supernatant of splenocytes co-cultured with GSLs-liposomes-loaded BMDCs, as compared to 88.33 ± 27.5 pg/mL of IFN-γ in the supernatant of splenocytes co-cultured with free GSLs-loaded BMDCs (Figure 2A). On the contrary, the IL-4 level was to be 269.7 ± 22.8 pg/mL in the supernatant of splenocytes co-cultured with free GSLs-loaded BMDCs (Figure 2B), as compared to 225.3 ± 35.2 pg/mL in the supernatant of splenocytes co-cultured with GSLs-liposomes-loaded BMDCs (Figure 2B).

### 2.3. Immunization with WCAgs-GSLs-Liposomes or WCAgs-Liposomes Induced No Toxicity

**Figure 3:**
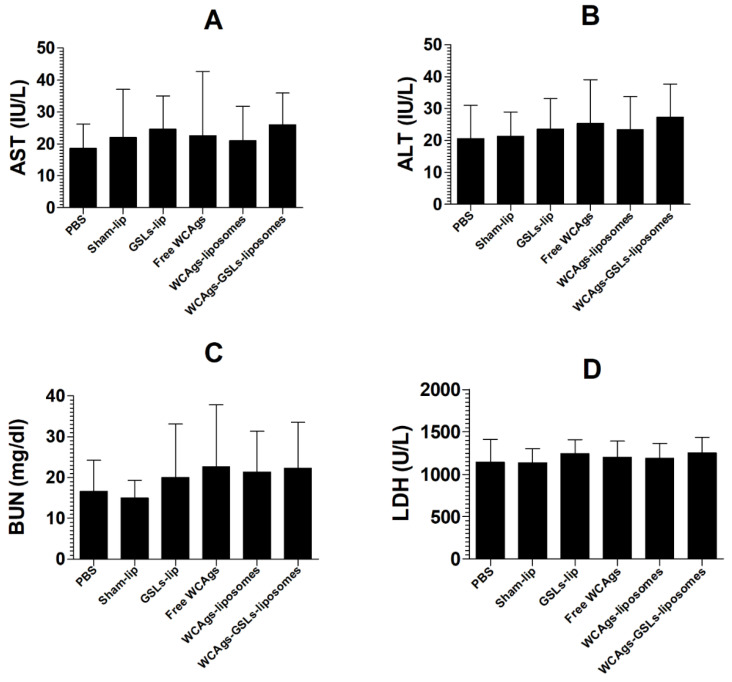
WCAgs-GSLs-liposomes did not induce any toxicity in mice. On day 5 post-final booster dose, the blood was collected to analyze the levels of (**A**) AST, (**B**) ALT level, (**C**) BUN, and (**D**) LDH. The data are presented as the mean ± SD of two different experiments.

Vaccination with free WCAgs, WCAgs-liposomes, or WCAgs-GSLs-liposomes was found to be safe, since the immunized mice did not demonstrate toxic manifestations and showed normal levels of enzyme parameters in the blood (Figure 3). The AST was found to be 26 ± 4 IU/L in WCAgs-GSLs-liposomes-immunized mice, whereas it was estimated to be 21 ± 4.4 IU/L in the mice immunized with WCAgs-liposomes (Figure 3A). These values were comparable to 22 ± 6 IU/L in sham liposomes-injected mice. Similar to AST, the ALT level was not altered significantly, as the mice immunized with WCAgs-GSLs-liposomes had an ALT value of 27 ± 4.2 IU/L, whereas those immunized with WCAgs-liposomes had 23 ± 4.1 IU/L (Figure 3B). The level of BUN was determined as the renal toxicity marker in immunized mice. It was found to be 22.3 ± 4.5 mg/dL in the blood of WCAgs-GSLs-liposomes-immunized mice, whereas the level of BUN was 21.3 ± 4 mg/dL in WCAgs-liposomes-immunized mice (Figure 3C). Similarly, the LDH level was analyzed as the cardiac toxicity parameter in immunized mice. Mice vaccinated with WCAgs-GSLs-liposomes had the LDH level of 1249 ± 74 U/L, whereas those immunized with WCAgs-liposomes had 1189 ± 71 U/L (Figure 3D).

### 2.4. WCAgs-GSLs-Liposomes Induced the Higher Secretion of IgG and Lymphocyte Proliferation

**Figure 4:**
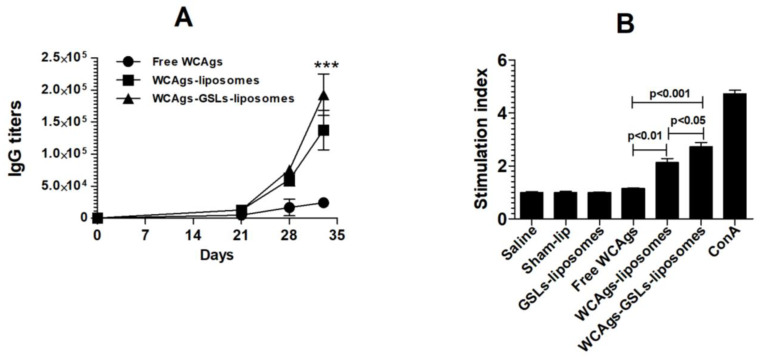
Mice immunized with WCAgs-GSLs-liposomes showed the highest IgG secretion and lymphocyte proliferation. The levels of (**A**) IgG, *** (*p* < 0.001) WCAgs-GSLs-liposomes vs. WCAgs-liposomes or free WCAgs (**B**) lymphocyte proliferation. The data are represented as the mean ± SD of three values.

Immunization with WCAgs-GSLs-liposomes resulted in the higher production of antigen-specific IgG, as compared to those with free WCAgs or WCAgs-liposomes, particularly on day 33 (Figure 4A) (*p* < 0.001). Immunization with free WCAgs (without any adjuvant) was ineffective in inducing the higher level of IgG production. Remarkably, WCAgs-GSLs-liposomes comprised the most effective vaccine formulation to elicit the highest production of IgG, followed by WCAgs-liposomes.

The effect of immunization with free WCAgs, WCAgs-liposomes, or WCAgs-GSLs-liposomes was also investigated by analyzing the splenocytes proliferation. The splenocytes from WCAgs-GSLs-liposomes-immunized mice showed the greatest proliferation among all vaccine formulations used in this study (Figure 4B). The stimulation index of splenocytes from the mice immunized with WCAgs-GSLs-liposomes was found to be 2.73 ± 0.277, as compared to the stimulation index of 2.14 ± 0.25 of the splenocytes from WCAgs-liposomes-immunized mice (Figure 4B).

### 2.5. WCAgs-GSLs-Liposomes-Immunized Mice Had Stimulated Immune Responses, in Terms of Cytokines and Co-Stimulatory Molecules

**Figure 5:**
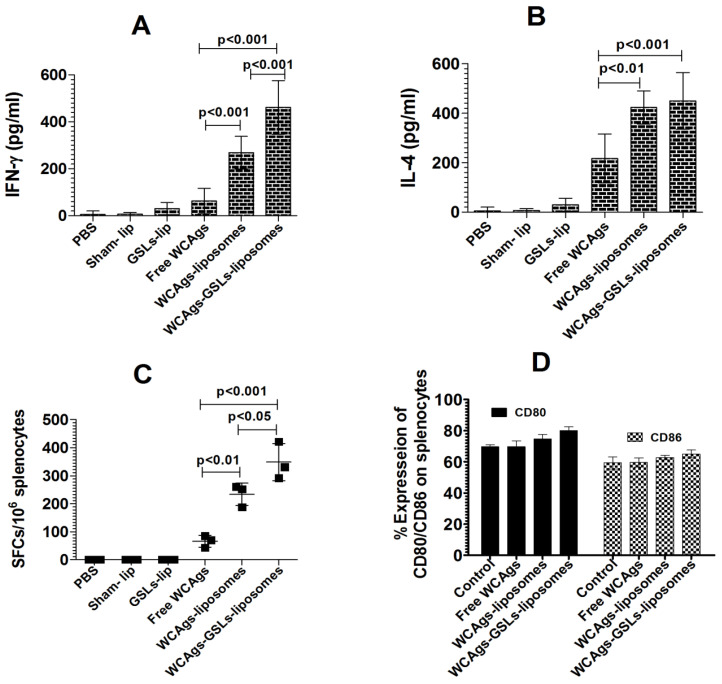
Immunization with WCAgs-GSLs-liposomes elicits greater immune responses. (**A**) IFN-γ, and (**B**) IL-4. The cells were exposed to WCAgs at a dose of 10 µg/mL. After 48 h, the amounts of IFN-γ and IL-4 were analyzed in the supernatant. (**C**) The frequency of IFN-γ producing splenocytes was determined by using an ELISPOT assay. (**D**) The splenocytes (5 × 10^5^) from the mice of various immunized groups were treated with FITC-conjugated anti-CD11c and PE-conjugated anti-CD80/CD86 for 1 h. The cells were washed, fixed, and analyzed by flow cytometry. The data are representative of two independent experiments.

The production of IFN-γ and IL-4 was measured in the supernatant of the splenocytes from mice immunized with free WCAgs, WCAgs-liposomes, or WCAgs-GSLs-liposomes (Figure 5). The amount of IFN-γ produced by the splenocytes from WCAgs-GSLs-liposomes-immunized mice was 462 ± 46 pg/mL, whereas the mice immunized with WCAgs-liposomes secreted 269 ± 28 pg/mL of IFN-γ (Figure 5A) (*p* < 0.001). However, the immunization with free WCAgs resulted in the lowest secretion of IFN-γ (63 ± 22 pg/mL). Besides IFN-γ, the amount of IL-4 was also quantified in the supernatant of the cultured splenocytes. The splenocytes from WCAgs-GSLs-liposomes-immunized mice secreted 450 ± 46 pg/mL of IL-4 (Figure 5B), whereas the mice immunized with WCAgs-liposomes produced 423 ±27 pg/mL of IL-4. However, free WCAgs-immunized mice produced 217 ± 40 pg/mL of IL-4 level.

Immunization with WCAgs-GSLs-liposomes induced the production of IFN-γ producing cells, as determined by the ELISPOT assay (Figure 5C). The numbers of SFCs were found to be 348 ± 56 per 1 × 10^6^ splenocytes in mice immunized with WCAgs-GSLs-liposomes, as compared to 237 ± 49 SFCs in WCAgs-liposomes-immunized mice (*p* < 0.05). Mice immunized with free WCAgs had only 66 ± 23 SFCs. The stimulation of the splenocytes with ovalbumin (used as non-specific antigen) did not induce any detectable IFN-γ production.

The effect of immunization with free WCAgs, WCAgs-liposomes, or WCAgs-GSLs-liposomes was assessed by analyzing the expression of CD80 and CD86 on the splenic DCs (Figure 5D). The percent expression of CD80 on splenic DCs from WCAgs-GSLs-liposomes-immunized mice was found to be 80 ± 1.0, as compared to 74.6 ± 1.1 and 69.6 ± 1.5 on the splenic DCs from free WCAgs and WCAgs-liposomes-immunized mice, respectively (Figure 5D). Similar to CD80, CD86 expression was also higher on the splenic DCs from WCAgs-GSLs-liposomes-immunized mice (65 ± 1.0), as compared to those immunized with free WCAgs (62.6 ± 0.58) and WCAgs-liposomes (57.7 ± 1.1). However, there was no significant difference in the cell surface expression of MHC class I, class II, and CD1d molecules on the splenocytes.

### 2.6. Anti-sera from WCAgs-GSLs-Liposomes-Immunized Mice Effectively Inhibited the Formation of Biofilm

**Figure 6:**
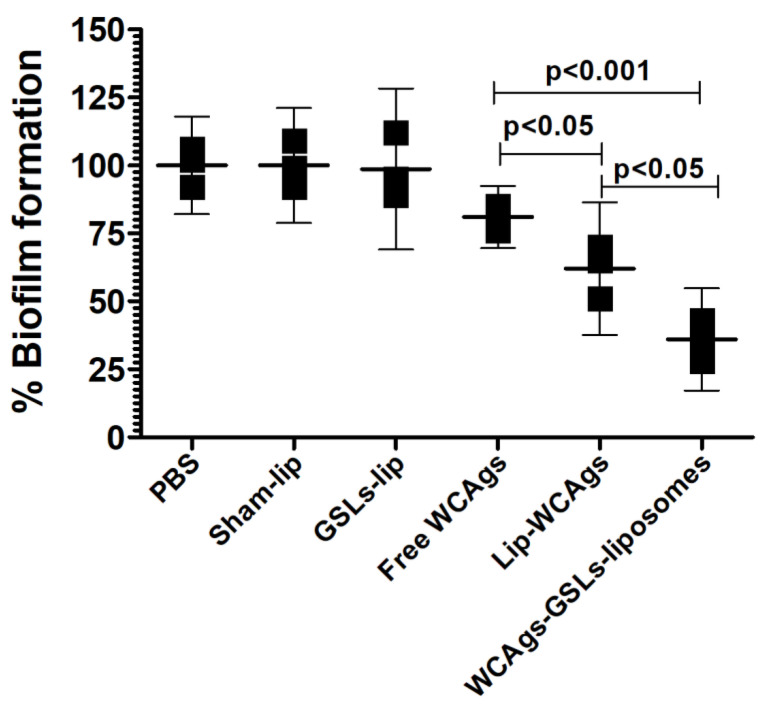
The anti-sera from the mice immunized with WCAgs-GSLs-liposomes were the most effective in inhibiting the biofilm formation by *A. baumannii*. The data are shown as the mean ± SD of three values.

The activity of the sera from the mice immunized with free WCAgs, WCAgs-liposomes, or WCAgs-GSLs-liposomes was analyzed against biofilm formation by *A. baumannii* (Figure 6). Anti-sera from WCAgs-GSLs-liposomes showed the highest activity against biofilm formation and reduced it to 36%, as compared to the sera from unimmunized control mice, whereas the sera from WCAgs-liposomes-immunized mice decreased it to 62% (Figure 6) (*p* < 0.05). However, the sera from free WCAgs-immunized mice exhibited the lowest efficacy against the biofilm formation and lowered it to 81% of the sera of the control mice (Figure 6).

### 2.7. WCAgs-GSLs-Liposomes-Immunized Mice Showed Higher Resistance to A. baumannii

**Figure 7:**
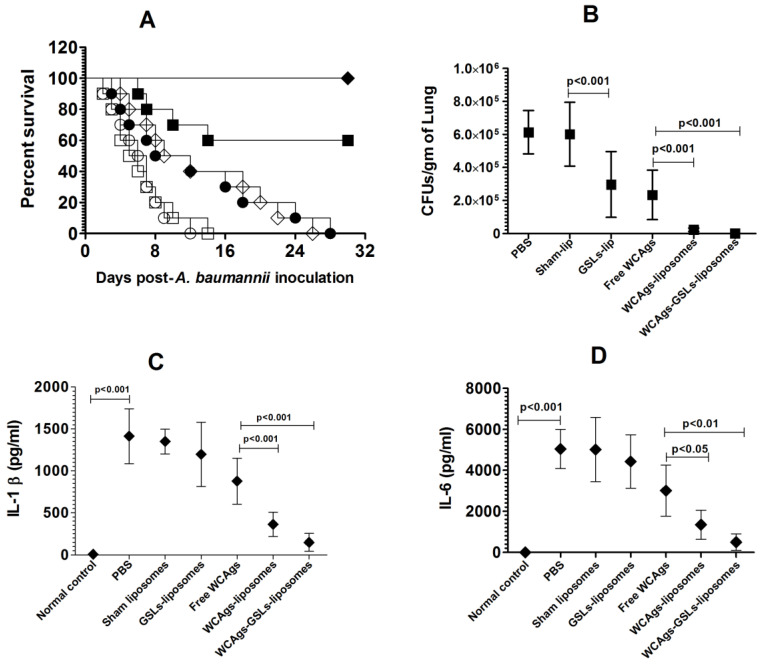
Vaccination with WCAgs-GSLs-liposomes effectively protected mice against *A. baumannii*. (**A**) Each control or immunized mouse was infected with 5 × 10^6^ bacterial cells and monitored daily for 30 days. PBS (◯), Sham-lip (☐), GSLs-lip (◇), free WCAgs (●), WCAgs-liposomes (■), WCAgs-GSLs-liposomes (♦). WCAgs-GSLs-liposomes vs. WCAgs-liposomes (*p* = 0.0291), WCAgs-GSLs-liposomes vs. free WCAgs (*p* < 0.001), WCAgs-liposomes vs. free WCAgs (*p* = 0.0102). (**B**) The bacterial load was analyzed in the lung tissues, as described in the methodology. (**C**) IL-1β and (**D**) IL-6 levels in BAL fluid of the control and immunized mice. The values are expressed as the means ± SD.

The prophylactic efficacy of immunization with WCAgs-GSLs-liposomes, WCAgs-liposomes, or free WCAgs was assessed against *A. baumannii* infection in a mouse model. Vaccination with WCAgs-GSLs-liposomes effectively protected the mice against *A. baumannii* infection, and the immunized mice had 100% survival rate (Figure 7A), which was significantly greater, as compared to 60% survival rate in mice vaccinated with WCAgs-liposomes (*p* = 0.0291). However, immunization with free WCAgs was ineffective, and all mice died within day 30 post-infection. Interestingly, the administration of GSLs-liposomes increased the median survival time of infected mice to 10.5 days, as compared to the 5.5 days of those injected with sham-lip (*p* = 0.017).

The findings of the survival data were also supported by the bacterial load in the lung tissues (Figure 7B). The mice in the PBS- and sham liposomes-injected groups had 613,819 ± 52,710 and 601,871 ± 77,693 CFUs/gram of the lung tissue, respectively, whereas mice injected with GSLs-liposomes contained 297,142 ± 80,224 CFUs/gram (Figure 7B) (*p* < 0.001). Mice immunized with WCAgs-GSLs-liposomes had the lowest bacterial load of 245 ± 136 CFUs/gram, whereas the mice vaccinated with WCAgs-liposomes possessed 22,722 ± 4059 CFUs/gram. In contrast, free WCAgs-immunized mice had 234,089 ± 60,539 CFUs/gram, which was significantly lower, as compared to the bacterial burden in the lung tissues of the mice immunized with WCAgs-GSLs-liposomes or WCAgs-liposomes (*p* < 0.001). The severity of the infection was also assessed by analyzing the levels of inflammatory cytokines, including IL-1β and IL-6, in the BAL fluid of the lungs of *A. baumannii*-infected mice (Figure 7C,D). The amount of IL-1β was found to be 1413 ± 171 pg/mL in un-immunized mice infected with *A. baumannii*. Immunized mice showed relatively reduced levels of IL-1β in their BAL fluid. Mice immunized with free WCAgs had 877 ± 110 pg/mL, which was further reduced to 358 ± 62 pg/mL in the BAL fluid of WCAgs-liposomes-immunized mice and to 148 ± 42 pg/mL in the BAL fluid of WCAgs-GSLs-liposomes-immunized mice (Figure 7C). Similar to IL-1β, the IL-6 was alleviated in immunized mice (Figure 7D). The amount of IL-6 was found to be 1413 ± 171 pg/mL in the BAL fluid of un-immunized mice infected with *A. baumannii*, 5045 ± 385 pg/mL in un-immunized mice, which was reduced to 3011 ± 502 pg/mL in free WCAgs-immunized mice, 1342 ± 286 pg/mL in WCAgs-liposomes-immunized mice, and 498 ± 160 pg/mL in WCAgs-GSLs-liposomes-immunized mice (Figure 7D).

### 2.8. Immunization with WCAgs-GSLs-Liposomes Showed Greater Efficacy against A. baumannii Infection in Immunocompromised mice

**Figure 8:**
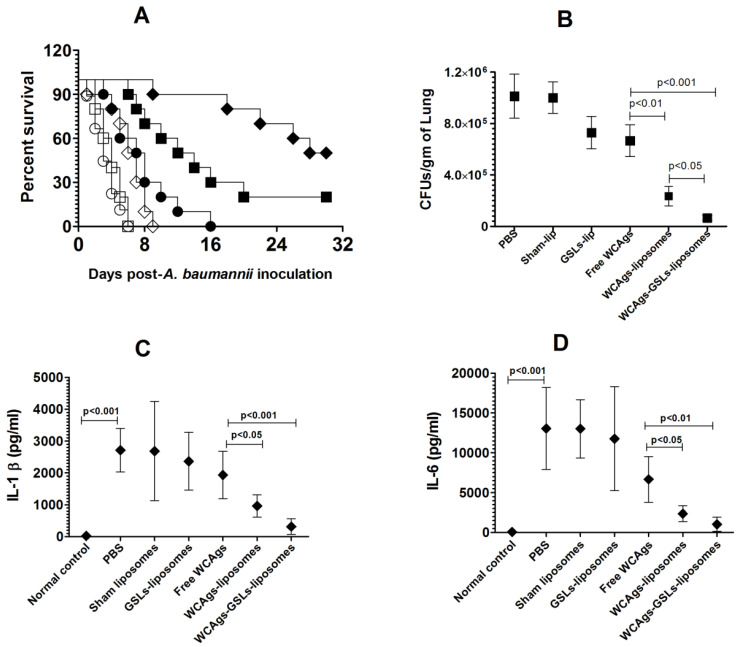
Immunization with WCAgs-GSLs-liposomes showed superior prophylactic potential against *A. baumannii* in immunocompromised mice. (**A**) Each control or immunized mouse was infected with 5 × 10^6^ bacterial cells and monitored daily for 30 days. PBS (◯), Sham-lip (☐), GSLs-lip (◇), free WCAgs (●), WCAgs-liposomes (■), WCAgs-GSLs-liposomes (♦). WCAgs-GSLs-liposomes vs. WCAgs-liposomes (*p* = 0.0388), WCAgs-GSLs-liposomes vs. free WCAgs (*p* < 0.001), WCAgs-liposomes vs. free WCAgs (*p* = 0.0228). (**B**) The bacterial load was analyzed in the lung tissues, as described in the methodology section. (**C**) IL-1β and (**D**) IL-6 levels in BAL fluid of the control and immunized mice. The values are expressed as the means ± SD.

The effectiveness of immunization with WCAgs-GSLs-liposomes, WCAgs-liposomes, or free WCAgs against *A. baumannii* was also evaluated in immunocompromised mice. Immunocompromised mice in the group immunized with WCAgs-GSLs-liposomes demonstrated the highest resistance and had a survival rate of 50% on day post-infection, whereas those immunized with WCAgs-liposomes exhibited a 20% survival rate (Figure 8A) (*p* = 0.0388). However, all the mice immunized with free WCAgs died within day 30 post-infection.

The enormity of infection was analyzed by assessing the bacterial load in lung tissues. The lung tissues from the mice immunized with WCAgs-GSLs-liposomes contained the lowest CFUs of *A. baumannii*, followed by those immunized with WCAgs-liposomes (Figure 8B). The lungs from WCAgs-GSLs-liposomes-immunized mice had 66,521 ± 18,716 CFUs/gram, whereas those immunized with WCAgs-liposomes contained 236,056 ± 76,881 CFUs/gram in the lung tissue (*p* < 0.05). The severity of *A. baumannii* infection was found to be highest in free WCAgs immunized mice among all immunized groups, as free WCAgs immunized mice had 667,422 ± 123,841 CFUs/grams in the lung tissue, which were significantly higher, as compared to CFUs numbers in the lung tissues of WCAgs-liposomes- or WCAgs-GSLs-liposomes-immunized mice (*p* < 0.01 and *p* < 0.001, respectively). In order to assess the prophylactic effect of immunization, the statuses of IL-1β and IL-6 were analyzed in the BAL fluid of *A. baumannii*-infected mice (Figure 8C,D). The amounts of IL-1β and IL-6 were found to be higher in the BAL fluid of immunocompromised mice, as compared to those in immunocompetent mice (Figure 7C, D and Figure 8C,D). The amount of IL-1β was found to be 2714 ± 275 pg/mL in PBS (un-immunized)-injected immunocompromised mice infected with *A. baumannii*. Immunized mice showed highly reduced amounts of IL-1β in BAL fluid. In particular, the immunocompromised mice immunized with free WCAgs contained 1936 ± 300 pg/mL of IL-1β in the BAL fluid, which was further decreased to 962 ± 142 pg/mL in WCAgs-liposomes-immunized mice and to 314 ± 98 pg/mL in WCAgs-GSLs-liposomes-immunized mice (Figure 8C). Similar to IL-1β, the amount of IL-6 was also found to be highly reduced in immunized mice (Figure 8D). The amount of IL-6 was found to be 13,045 ± 2070 pg/mL in the BAL fluid of PBS injected mice infected with *A. baumannii*. Immunocompromised mice immunized with free WCAgs contained 6668 ± 1155 pg/mL in the BAL fluid, whereas the mice immunized with WCAgs-liposomes had 2357 ± 405 pg/mL, and those immunized with WCAgs-GSLs-liposomes contained 1031 ± 356 pg/mL (Figure 8D).

### 2.9. Mice Immunized with WCAgs-GSLs-Liposomes Exhibited Less Severe Complications in the Lung Tissues

**Figure 9:**
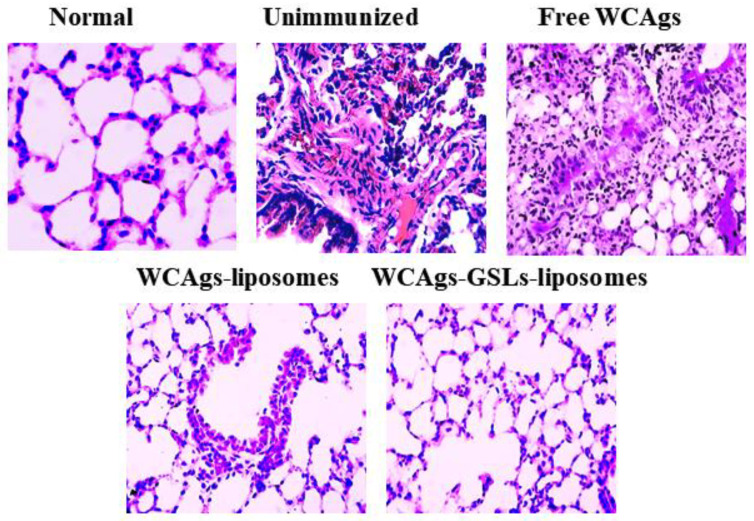
The immunocompromised mice vaccinated with WCAgs-GSLs-liposomes showed highly improved structural integrity in their lung tissues, as compared to the lung tissues from the unimmunized or free WCAgs- or WCAgs-liposomes-immunized mice.

*A. baumannii* infection induces robust inflammatory responses that impair the functioning of the lung tissues. The histological analysis revealed that unimmunized (PBS or sham-liposomes injected) mice showed the highest intensity of infection-associated complications, including massive hemorrhage, congestion, airway wall thickening, edema, and the infiltration of leukocytes, in comparison to the lung tissues from the normal mice (Figure 9). Anyway, extensive tissue fibrosis and the loss of the cellular integrity were also noticed (Figure 9). Interestingly, the lung tissues from WCAgs-GSLs-liposomes-immunized mice showed significantly alleviated pneumonia-associated changes, as compared to those from free WCAgs- or WCAgs-liposomes-immunized mice (Figure 9).

## 3. Materials and Methods

### 3.1. Materials

HPLC-grade chloroform and methanol were bought from Thermo-Fisher Scientific (Waltham, MA, USA). Liposome-grade lipids, including DPPC, cholesterol, kits for cytokines, inflammatory markers, or cell proliferation assay, and antibodies for MHC class I, class II, and CD1d, were purchased from the Abcam (Cambridge, UK). Serum alanine transaminase (ALT), aspartate transaminase (AST), blood urea nitrogen (BUN), and lactate dehydrogenase (LDH) kits were purchased from Quimica Clinica Aplicada (Amposta, Tarragona, Spain). The isotypes analysis kits were purchased from the Sino Biologicals Inc. (Beijing, China). The status of NKT cells was investigated by staining the splenocytes using mCD1d/α-GalCer-loaded tetramer (tetramer core faculty of the National Institute of Health, Emory University, Atlanta, GA, USA).

### 3.2. Sphingomonas paucimobilis

*Sphingomonas paucimobilis* was bought from the American Type Culture Collection (ATCC 10829, Manassas, VA, USA).

### 3.3. Preparation of Whole Cell Antigens (WCAgs) from A. baumannii

A MDR strain of *A. baumannii* (ATCC 19606) was cultured in tryptic soya broth (TSB) for 24 h. The culture was centrifuged, and the bacterial pellet was collected, washed, and taken into lysis buffer (0.75 M sucrose, 10 mM Tris-HCl, 10 mg/mL of lysozyme, and 1.5 mM EDTA and protease inhibitor cocktail). The cell lysate was sonicated and centrifuged at 7800× *g*, in order to collect the supernatant that was used to determine protein concentration by BCA method [24].

### 3.4. Isolation of GSLs from S. paucimobilis

Glycosphingolipids (GSLs) were isolated from *S. paucimobilis*, as described in our earlier reported [23]. Briefly, *S. paucimobilis* was grown in nutrient broth, and the media was centrifuged, in order to save cell pellets. After washing, the bacterial pellet was lyophilized, followed by homogenization in a mixture of methanol and chloroform (2:1). The lipid extract was separated from the non-lipid residue that was re-extracted with chloroform/methanol mixture (1:1, vol:vol), as above. The lipid extracts were combined and dried by flash evaporation under vacuum. Chloroform: methanol (2:1, vol:vol)-soluble fraction was used as a crude lipid extract. In order to remove the phospholipids, the crude lipid extract was reconstituted with the help of warm methanol containing 0.5 N KOH at 37 °C for 4 h. 

### 3.5. Preparation of Antigen-Loaded GSLs-Free and GSLs-Bearing Liposomes

GSLs-bearing or GSLs-free liposomes were prepared as described earlier [23]. DPPC and cholesterol (with or without GSLs) in the molar ratios of 10:6 were dissolved in chloroform and methanol. The solvents were evaporated using a stream of nitrogen gas that results in the formation of a lipid film on the wall of the flask. The film was hydrated, and the suspension was sonicated by a probe sonicator. WCAgs was added to lipid suspension, and the mixture was lyophilized to form dried and reconstituted vesicles (DRVs). The DRVs were rehydrated, and the suspension was centrifuged for 20 min at 4 °C. The quantity of WCAgs in GSL-free and GSL-bearing liposomes was estimated, and the amount of encapsulated antigen was calculated as follows: 1-A_Free antigen_/A_Total antigen._ The percentages of encapsulation efficiency (%EE) of WCAgs were found to be 54% and 52% in GSL-free and GSL-bearing liposomes, respectively. 

The characteristics of liposomes, such as the polydispersity index (PDI), size, and zeta potential, were determined by the Malvern nano zetasizer by using the dynamic light scattering (DLS) technique [23].

### 3.6. Mice

Female BALB/C mice (12 weeks) were taken from the animal house facility of the College of Applied Medical Sciences, Qassim University. Mice were housed in pathogen-free conditions and monitored two times daily throughout the duration of the study. Studies in animal were approved by the committee of research ethics, Deanship of Scientific Research, Qassim University, Buraydah, Saudi Arabia.

### 3.7. Isolation of Bone Marrow-Derived Dendritic Cells (BMDCs) and Loading with GSLs-Liposomes

BMDCs were made by flushing the femur bones and were cultured in RPMI-1640 media containing 2 mm L-glutamine, 50 µM 2-mercaptoethanol, 10% FBS, and murine GM-CSF (10 ng/mL) in T75 cell culture flask [23]. After 6 days, the loosely attached BMDCs were collected, and 1 × 10^5^ cells per well (96 well plate) were incubated with free GSLs or GSL-bearing liposomes (100 µg/mL of GSLs) or sham liposomes (no GSLs) for 12 h. 

Splenocytes (5 × 10^5^ cells) isolated from the fresh mice were incubated with GSLs-treated BMDCs. After 48 h, the incubation mixture was centrifuged, and the supernatant was used to analyze the status of IFN-γ and IL-4.

### 3.8. Immunization of Mice with WCAgs-Liposomes and WCAgs-GSLs-Liposomes

**Figure 10:**
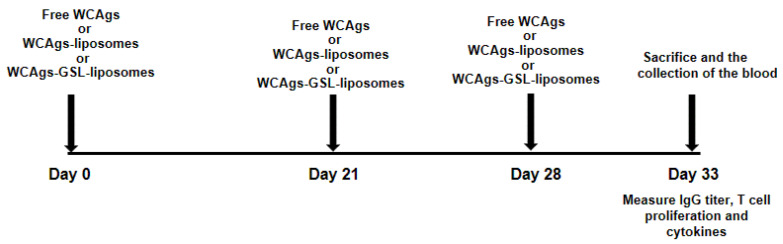
Schematic illustration of the immunization schedule in immunocompetent mice.

Each mouse was immunized with WCAgs-liposomes, WCAgs-GSLs-liposomes, or free WCAgs (20 µg) through the subcutaneous route. The booster doses were injected on days 21 and 28 (Figure 10). There were six groups of mice, and each contained 10 mice.

1. PBS;
2. Sham liposomes;
3. GSLs-liposomes;
4. Free WCAgs;
5. WCAgs-liposomes;
6. WCAgs-GSLs-liposomes.

### 3.9. Analysis of Total IgG and Lymphocyte Proliferation

The effect of immunization with free WCAgs, WCAgs-liposomes, or WCAgs-GSL-liposomes on antigen-specific immune responses were determined by analyzing antibody titer and lymphocyte proliferation [24]. On day 5, after the final booster dose, the blood was collected to quantify the total IgG.

In order to analyze the effect of immunization on lymphocyte proliferation, the spleen was removed and homogenized to prepare splenocytes. The proliferation assay was achieved by using cell titer 96 non-radioactive BrdU calorimetric ELISA kit from Abcam (Cambridge, UK). Splenocytes (1 × 10^5^) were stimulated with 10 µg/mL of WCAgs for 48 h, whereas the cells stimulated with Con A and ovalbumin acted as the positive and negative controls, respectively.

### 3.10. ELISPOT Assay

The ELISPOT assay was performed to analyze IFN-γ producing splenocytes from the immunized mice. The ELISPOT plates were pre-coated with anti-IFN-γ, followed by the addition of 5 × 10^5^ splenocytes/well. Next, 20 µg/mL of WCAgs or ovalbumin (as a control) was incubated with the splenocytes. After 48 h of incubation, the medium was aspirated, and 100 µL of biotinynated anti-IFN-γ was added to each well for next 2 h. Followed by washing, 100 µL of streptavidin-alkaline phosphatase mixture was added and incubated for 1 h at room temperature. In order to analyze the spot formation, BCIP/NBT substrate (100 µL/well) was added, and the plates were used to count the numbers of spot forming cells (SFCs).

### 3.11. Analysis of IFN-γ and IL-4

The splenocytes (1 × 10^6^) from various groups of mice were stimulated with 10 µg/mL of WCAgs and incubated for 48 h at 37 °C. As a control, the splenocytes were treated with 10 µg/mL of ovalbumin. The supernatant was collected to determine the levels IFN-γ and IL-4.

### 3.12. Determination of the Expression of Co-Stimulatory Molecules and antigen Presenting Molecules in Splenocytes

The splenocytes (5 × 10^5^) pre-incubated with rat anti-mouse CD16/CD32 (used as Fc blockers) were treated with FITC-conjugated anti-CD11c and PE-conjugated anti-CD80/CD86 for 1 h. The cell surface expression of MHC class I, class II, and CD1d molecules was analyzed on the splenocytes from various groups of mice. The splenocytes were stained with PE-conjugated anti-MHC I, class I, and CD1d antibodies. The cells were washed and fixed in 1% paraformaldehyde for 15 min. After washing, the cells were suspended in HBSS-BSA and analyzed by flow cytometry.

### 3.13. To Determine the Effect of Anti-Sera on the Formation of Biofilm by A. baumannii

The anti-sera from the mice of various immunized groups were tested to analyze their effects on the biofilm formation [25]. *A. baumannii* (5 × 10^5^ cells/mL) in nutrient broth (NB) was incubated with 10-fold diluted anti-sera for 24 h. After washing with PBS, the wells were dried and stained with 0.1% crystal violet solution for 10 min. The wells were washed and dried, followed by the addition of 150 µL of ethanol. Finally, the absorbance was recorded at 595 nm by using a microplate spectrophotometer.

### 3.14. Infection of Mice with A. baumannii

*A. baumannii* cells grown in NB were centrifuged, and the bacterial cell pellet was washed twice with sterile PBS. Each mouse was infected with 5 × 10^6^ CFUs of *A. baumannii* through the intravenous route 7 days after the final booster dose.

### 3.15. To Determine the Prophylactic Efficacy of Vaccine Formulations against A. baumannii

The efficacy of vaccine formulation against *A. baumannii* infection was assessed by monitoring the data of survival rate and bacterial load [25]. In order to analyze the survival rate, the mice were observed two times a day for 30 days, whereas the bacterial burden was analyzed in the lung tissues of mice. On day 5 post-infection, three mice from each group were sacrificed, and the lungs were excised and homogenized in cold-PBS. Various dilutions of tissue homogenates were plated on nutrient agar in triplicates. After 24 h, the colony forming units (CFUs) were counted, and the numbers were multiplied by the dilution factor.

### 3.16. Immunization of Immune-Compromised Mice

**Figure 11:**
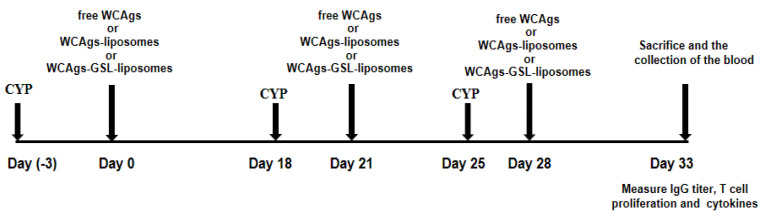
Schematic illustration of the immunization schedule in immunocompromised mice.

Cyclophosphamide (CYP) at a dose of 100 mg/kg were injected 3 days before each dose of immunization, as described in our earlier study [26] (Figure 11). Mice were divided into the following groups, and each group contained 12 mice.

### 3.17. Infection of Immunocompromised Mice with A. baumannii

*A. baumannii* (5 × 10^6^ CFUs) were injected into each mouse 7 days post-final booster dose.

### 3.18. Effectiveness of Vaccine Formulations in Protection against A. baumannii in Immunocompromised Mice

The potential of vaccine formulations to protect against *A. baumannii* was investigated in immunocompromised mice by examining the survival rate and bacterial load data [25]. The survival rate was assessed by monitoring the mortality of mice over 30 days post-infection. The severity of the infection was determined by assessing the bacterial load in the lung tissues and analyzing inflammatory cytokines in BAL fluid of the mice.

### 3.19. Histological Analysis of Lung Tissues

The intensity of *A. baumannii* infection-associated pathological alterations were examined by the histological study of the lung tissues as described earlier [27]. The lungs were harvested and fixed into 10% neutral-buffered formalin solution. The sections of tissues measuring 5 μm thicknesses were sliced by using paraffin embedded blocks. The slides containing the tissues were stained with a hematoxylin–eosin solution. The tissues were examined under a light microscope to detect the pathological changes.

### 3.20. Statistical Analyses

The data of antibody, cytokines, CD80, CD86, and biofilm inhibition were analyzed by one-way ANOVA and Tukey’s post-hoc test by using the GraphPad Prism software, version 5.0 (La Jolla, CA, USA). The survival data was analyzed by the Kaplan–Meier curve and analyzed by the log-rank Chi square test.

## 4. Discussion

*A. baumannii* has been a leading pathogen that caused about 50,000 deaths in 2019, due to its ability to resist multiple antibiotics [28]. Due to the ineffectiveness of a majority of presently available antibiotics, the preparation of an effective vaccine formulation against *A. baumannii* becomes critical as a prophylactic measure to prevent the seriousness of the infection. Multiple vaccine antigens, such as outer membrane protein A (OmpA), outer membrane complexes (OMCs), inactivated whole cells, and biofilm-associated protein (Bap), have been recognized as immunogenic molecules that elicit antigen-specific immune responses [29,30,31,32,33,34,35,36]. Some of them exhibited weak immunogenicity, whereas others have safety issues. Unfortunately, none of these formulations advanced to the clinical stage.

Liposomes act as potent immunoadjuvant and antigen-delivery systems that can elicit the humoral and cell-mediated immune responses [37,38]. The modification of liposomes can enhance the immunogenicity of the encapsulated antigens, thus resulting in the desired immune responses [39]. Here, we isolated GSLs from *S. paucimobilis* and incorporated them into liposomes that were used as a vaccine delivery system for WCAgs of *A. baumannii*. Immunization with WCAgs-GSLs-liposomes induced strong humoral and cell-mediated immune responses in vaccinated mice. The determination of the safety of a vaccine formulation is the first important step to apply in clinical trials. The safety profiles of WCAgs-liposomes and WCAgs-GSLs-liposomes were tested by analyzing the status of AST, ALT, BUN, and LDH in the blood of immunized mice. Importantly, both WCAgs-liposomes and WCAgs-GSLs-liposomes vaccine formulations were found to be safe, as they did not cause any toxicity in immunized mice.

The immunogenic potential of vaccine formulations were evaluated by measuring the levels of antibodies and lymphocyte proliferation. The data showed that mice immunized with WCAgs-GSLs-liposomes had greater levels of antibodies, in comparison to those immunized with WCAgs-liposomes or free WCAgs. Earlier researchers demonstrated that membrane proteins-specific IgG can opsonize the bacteria and protect the mice against multidrug-resistant isolates of *A. baumannii* [40,41]. Anyway, the elevated proliferation of lymphocytes was also observed in the splenocytes from the mice immunized with WCAgs-GSLs-liposomes. Moreover, the levels of IFN-γ and IL-4 were analyzed to evaluate the immune-potentiating effects of vaccine formulations. The mice immunized with WCAgs-GSLs-liposomes had significantly higher levels of these cytokines. Interestingly, the numbers of IFN-γ-producing cells were also found to be higher in the spleen of the mice immunized with WCAgs-GSLs-liposomes. The greater immune-stimulating potential of WCAgs-GSLs-liposomes can be attributed to the presence of GSLs, as the GSLs-loaded BMDCs stimulated the splenocytes to secrete higher cytokines, particularly IFN-γ. IFN-γ can activate the cell-mediated immunity by increasing the activity of NK cells, macrophages, and cytotoxic T lymphocytes. Furthermore, the splenic DCs from mice immunized with WCAgs-GSLs-liposomes exhibited a higher expression of co-stimulatory molecules, such as CD80 and CD86, that may contribute to the increased immunogenicity of WCAgs-GSLs-liposomes. The maturation of DCs also plays a very important role in increasing the immunogenicity of a vaccine antigen [42,43].

The biofilm formation by *A. baumannii* helps bacteria to thrive under hostile conditions and also contributes to multidrug resistance, as the biofilm matrix restricts the penetration of the antibiotics into the deeper layers [44,45]. Moreover, the drug efflux pump AdeABC has been shown to play a role in the formation of biofilm by *A. baumannii* [46]. The outer membrane protein A (ompA) and pili of *A. baumannii* also take part in the adherence of the biofilm to the surfaces [45,47]. The results of the present work demonstrated that anti-sera from the WCAgs-GSLs-liposomes-vaccinated mice effectively reduced the biofilm formation. It indicates that antibodies have the potential to inhibit the biofilm formation. Previous studies showed that antibodies can elevate the sensitivity of drug-resistant *A. baumannii* to antibiotics [48]. Another study by Nielson et al. showed that anti-*A. baumannii* monoclonal antibody showed a synergistic effect with colistin against bacterial sepsis and pneumonia [49].

In accordance with its effect on the antigen-specific immune response in mice, WCAgs-GSLs-liposomes formulation imparted the highest protection against *A. baumannii*. Mice immunized with WCAgs-GSLs-liposomes showed a 100% survival rate, whereas those immunized with WCAgs-liposomes (without GSLs) had a 60% survival rate. Since immunocompromised subjects are easy targets to *A. baumannii* infection, we tested the prophylactic efficacy of WCAgs-GSLs-liposomes or WCAgs-liposomes against *A. baumannii* infection in immunocompromised mice. The data showed that the immunocompromised mice immunized with WCAgs-GSLs-liposomes demonstrated a 50% survival rate, as compared to the 20% survival rate of mice in the group immunized with WCAgs-liposomes. The effectiveness of WCAgs-GSLs-liposomes, as an effective vaccine formulation, was also supported by the bacterial load data in the lung tissues of mice. Mice immunized with WCAgs-GSLs-liposomes had the lowest numbers of bacterial CFUs in their lung tissues. The data on survival and bacterial burden were in accordance with the status of inflammatory cytokines, such as IL-1β and IL-6, in the BAL fluid. We earlier that showed the enhanced levels of IL-1β and IL-6 in *A. baumannii*-infected mice [50]. The IL-1β contributes to the pathogenesis of *A. baumannii* infection because IL-1R-deficient mice had decreased severity in the lung tissues [51]. The findings of the current work demonstrated that there was a remarkable reduction in IL-1β in WCAgs-liposomes- and WCAgs-GSLs-liposomes-immunized mice. Similar to IL-1β, the IL-6 level was elevated in the BAL fluid of *A. baumannii*-infected mice. The higher level of IL-6 has been shown to play a role in infection-associated inflammation (51). Immunization with WCAgs-GSLs-liposomes showed remarkable protection against *A. baumannii*, as immunized mice showed a significantly lower IL-6 amount in their BAL fluid. Earlier researchers induced the establishment of *A. baumannii*-associated pneumonia in mice through the intranasal route of infection [52]. However, we successfully induced *A. baumannii*-associated pneumonia by infecting the mice through the intravenous route, as well [26]. The gravity of the *A. baumannii* infection was studied by the histological analysis of the lung tissues, in order to examine the involvement of the lungs. The lung tissues of immunocompromised mice lost their structural integrity and were highly infiltrated by the inflammatory cells [26]. Since immunization with WCAgs-GSLs-liposomes was found to be effective in eliminating *A. baumannii* infection of the lung tissues, this effect was also reflected in the findings of the histological analysis. The results demonstrated that lung tissues from WCAgs-GSLs-liposomes-immunized mice had the alleviated effects of the infection, with less congestion and much reduced infiltration of inflammatory cells.

In conclusion, the data of this study demonstrated that WCAgs-GSLs-liposomes showed its efficacy as an effective prophylactic vaccine formulation to protect both the immunocompetent and immunocompromised mice against *A. baumannii*. Importantly, a vaccine formulation of WCAgs-GSLs-liposomes or WCAgs-liposomes did not induce any toxicity; moreover, it elicited a higher production of antibodies and cytokines, particularly IFN-γ levels in the immunized mice. The splenocytes from the mice immunized with WCAgs-GSLs-liposomes had a higher expression of CD80 and CD86. Mice immunized with WCAgs-GSLs-liposomes showed the highest resistance to *A. baumannii*, as shown by the survival rate and bacterial load data. Interestingly, the vaccination with WCAgs-GSLs-liposomes was also found to be effective in immunocompromised mice, as the immunized mice showed greater survival rates and reduced severity of the infection in the lung tissues. These findings together support the use of WCAgs-GSLs-liposomes as a prospective prophylactic vaccine formulation against *A. baumannii* infection, particularly in immunocompromised subjects.

## Figures and Tables

**Table 1:** Characteristic of liposome formulations.

Liposomal Formulations	PDI	Size (nm)	Zeta Potential
Lip-WCAgs	0.286	110	−12.4
GSLs-lip-WCAgs	0.312	128	−16.6

## Data Availability

All relevant data have been provided within the manuscript.

## Sample Availability

Samples of the compounds are available from the authors.
